# Choroidal remodeling distribution pattern in the macular region in Chinese young patients with myopia

**DOI:** 10.1186/s12886-021-02139-3

**Published:** 2021-10-18

**Authors:** Jun Wang, Xin Ye, Xiangjun She, Jiahao Xu, Yiqi Chen, Jiwei Tao, Xinjie Ye, Lijun Shen

**Affiliations:** School of Ophthalmology and Eye Hospital, Wenzhou Medical University, 270 West Xueyuan Road, Wenzhou, Zhejiang China

**Keywords:** Young myopic eyes, Choroidal vascularity index (CVI), Choroidal thickness (CT), Spherical equivalent (SE), Axial length (AL)

## Abstract

**Background:**

The pathogenesis of myopia has been found to be associated with the blood supply of the choroid. This study aimed to determine the relationship between the distribution pattern of choroidal remodeling and the degree of myopia in young patients.

**Methods:**

Young patients (age < 18 years) with the spherical equivalent of less than − 12 diopters (D) were included. Spectral-domain optical coherence tomography (SD-OCT) with enhanced depth imaging (EDI) modality was used to measure the choroidal thickness (CT) and choroidal vascularity index (CVI) in the macular regions. CVI was calculated as the proportion of luminal area to choroidal area and was measured within 1 mm and 3 mm nasal (N1 and N3), temporal (T1 and T3), superior (S1 and S3), and inferior (I1 and I3) to the foveal center. CVI was compared across different ages (i.e., 5 ~ 9 years, 10 ~ 13 years, and 14 ~ 18 years), axial lengths (ALs) (i.e., 21.00 ~ 25.00 mm and 25.01 ~ 29.00 mm), and spherical equivalents (SEs) (i.e., SE > -0.5D, − 0.5 ~ − 3.0D, − 3.01 ~ − 6.0D, and < − 6.0D). Linear regression analysis was applied to assess the association between independent (i.e., age, AL, SE, and intraocular pressure) and dependent variables (i.e., CVI of different regions).

**Results:**

One hundred sixty-four eyes from 85 volunteers were included. The mean CT in the central foveal was 269.87 ± 63.32 μm (93.00 μm to 443.00 μm). The mean subfoveal-CVI was 67.66 ± 2.40% (57.84 to 79.60%). Multiple linear regression results revealed significant correlations between SE and T1-CVI (*p* < 0.05, *r*^2^ = 0.082, β = 0.194), N1-CVI (*p* < 0.05, *r*^2^ = 0.039, β = 0.212). Simple linear regression results revealed that T1-CVI (*p* < 0.05, *r*^2^ = 0.09) and T3-CVI (*p* < 0.05, *r*^2^ = 0.05) were negatively correlated with SE; N1-CVI (*p* < 0.05, *r*^2^ = 0.05) and N3-CVI (*p* < 0.05, *r*^2^ = 0.04) were negatively correlated with SE.

**Conclusions:**

CVI in the horizontal meridian underwent the largest change as myopia worsened. Temporal and nasal CVIs within the r = 1 mm, and r = 3 mm subfoveal range were positively associated with the degree of myopia in young patients. The CVI value may be used to assess the vascular status of the choroid and be a potential marker of myopic progression.

## Introduction

Myopia has become the second leading cause of blindness worldwide and can create burdens on individuals and society [1]. East Asian countries have experienced the fastest growth in cases of myopia. The prevalence of myopia among Chinese university students is about 95.5%, and high myopia accounts for about 19.5% [2]. A significant proportion of available research has been conducted in young myopic eyes, and long-lasting choroidal alterations have notable importance for myopic development [3, 4]. The choroid consists predominantly of blood vessels and is involved in numerous physiological processes of the eye [5]. Choroidal blood supply has been shown to be related to the pathogenesis of myopia [6], and choroidal thickness (CT) as well as choroidal vascularity index (CVI) are considered important measurement indexes for the status of choroid.

CT is defined as the perpendicular distance between the Bruch membrane and the choroid/sclera junction on the optical coherence tomography (OCT) images. In both normal and highly myopic eyes, CT values vary among different macula regions. CT is the thinnest at the nasal and inferior regions, followed by the superior and temporal regions [3, 7, 8]. Several studies have demonstrated a decreased CT in highly myopic eyes [9–11]. What is noteworthy is that Fang et al. [12] reported a CT cut-off value of 56.5 μm at 3 mm nasally to the fovea, which is suggested as a diagnostic criterion of pathological myopia. However, CT is affected by age, gender, axial length, intraocular pressure, systolic blood pressure [5]. Therefore, a more stable indicator needs to be found.

With the changes in CT, the subsequent remodeling of choroid vessels is worth studying. With an increase in luminal diameter, the choroid may be differentiated into choriocapillaris, Sattler’s layer, and Haller’s layer [13]. The choroidal stroma, which contains leukocytes, melanocytes, fibroblasts, and numerous non-vascular smooth muscle cells, fills extravascular and extra lymphatic space [14]. Unlike the lacunae action, with contraction of the non-vascular smooth muscle cells, the choroid is becoming thinning [14]. The CVI expressed as the proportion of luminal area to choroidal area, is a promising new parameter to assess the remodeling of choroid vessels and retinal blood supply in the macular area [15]. After binarization, the OCT image is transformed to a black-and-white one. The stroma or interstitial area is represented by light pixels, while the vessel lumens are indicated by dark pixels. Then CVI is derived by calculating the ratio between the luminal area and the choroidal area of the defined region. A recent study has noted that CVI was less influenced by physiologic factors mentioned above [16]. Thus, it is emerging as a potentially more robust biomarker for evaluating choroidal status in myopic progression.

The advancement of the spectral-domain optical coherence tomography (SD-OCT) renders possible the non-invasive and quantitative measurements of the choroid with the resolution of 12 *μ* m. In a clinical setting, enhanced depth imaging OCT (EDI-OCT) offers improvements in visualizing the choroid/sclera junction and observing the entire choroid. SD-OCT images can be processed for a detailed morphologic and vascular features analysis, including CT and the CVI at selected distances from the foveal center. Our study aimed to observe the distribution features of the CVI and CT in young patients by using SD-OCT with EDI technique to explore the characteristics of the distribution pattern of the choroidal remodeling during myopia progression.

## Methods

### Study population

The study included 172 eyes of 84 participants (aged 5–18 years) from the Zhejiang eye hospital and was approved by the Institutional Review Board. Informed written consent was obtained from every enrolled subject after a thorough explanation of the details of the study and potential risks and consequences of the study. Subjects were excluded if they suffered from corneal opacification, cataracts, glaucoma, retinal disease, or amblyopia.

### Comprehensive ophthalmic examinations

All participants went through comprehensive ophthalmic examinations, including the measurement of intraocular pressure (IOP), axial length (AL), cycloplegic refraction, slit-lamp, and dilated fundus examinations. For each participant, all tests were conducted within a day. IOP was measured three times by Goldmann applanation tonometry (GAT), and the average value was used as the outcome. The AL was measured by optical biometry (IOLMaster; Carl Zeiss, Jena, Germany). Their pupils were dilated with cyclopentane before the refraction test and fundus examination.

### Choroidal image acquisition

The choroidal thickness (CT) and choroidal vascularity index (CVI) in the macular region were examined by spectral-domain optical coherence tomography (SD-OCT, Optovue Inc., Fremont, CA, USA) with enhanced depth imaging (EDI) modality. Enhanced high-density B-scans (12 *μ* m resolution) were taken at different positions to obtain the choroidal images. The same trained technician performed horizontal and vertical scans crossing the fovea and the optic disk to ensure uniformity. Sixty scans at each position were montaged to create one image; only clear images were used for analysis. All the patients underwent the measurement in a seated position, and the EDI mode software (RTVue XR OCT Avanti System, version 2016.1.0; Optovue Inc., Fremont, CA, USA) was used to enhance the visibility of the choroidal structure [17].

### Choroidal thickness measurement

Subfoveal CT was manually measured by two independent researchers using the EDI-OCT, and the average thickness was considered the result. CT was calculated as the distance between the lower boundary of RPE and the choroid-scleral border. Measurements of both the right and left eyes of each subject were obtained and used for further analysis.

### Image binarization and choroidal vascularity index measurement

**Fig. 1:**
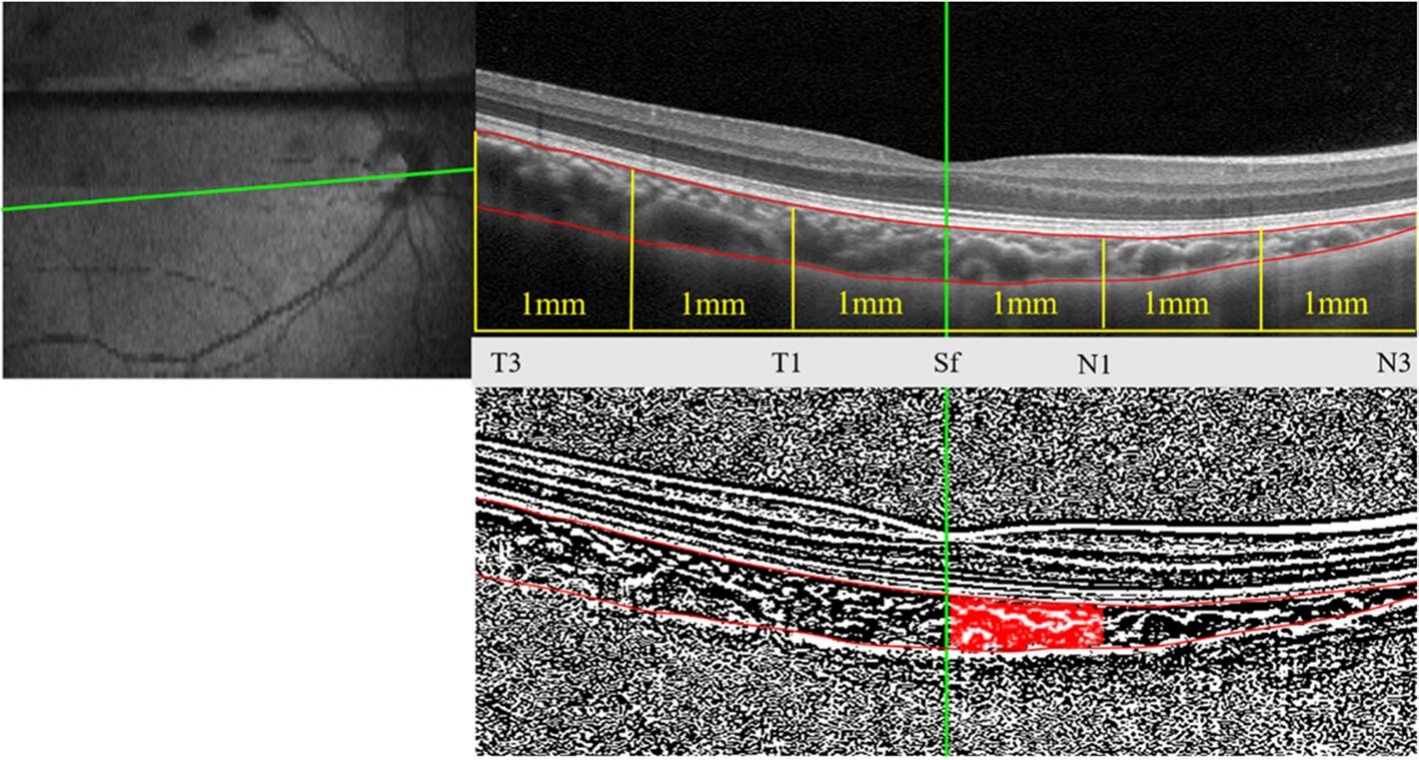
Image binarization for the choroid with normal choroidal thickness. **A** Original SD-OCT image; **B** 6 mm subfoveal choroidal area; **C** Segmented OCT image using modified image binarization approach. Overlay of the region of interest created after image binarization was performed on the SD-OCT image

All collected images were processed and analyzed using the public domain ImageJ software (freely available at http://imagej.nih.gov/ij/; National Institutes of Health [NIH], Bethesda, MD). The image was firstly binarized by Niblack autolocal threshold tool [18], which considers all the pixels’ mean and standard deviation(SD). After that, the choroid-scleral interface was clearly visualized and further enabled precise selection of the region of interest (i.e., total subfoveal choroidal area). The dark pixels representing the luminal area were selected by the color threshold tool, and residual pixels were considered as stroma area. From the collected choroidal images, CVI was calculated as the ratio of the vascular luminal area to the choroidal area, measured at subfoveal (Sf), 1 mm and 3 mm nasal (N1 and N3, respectively), temporal (T1 and T3, respectively), superior (S1 and S3, respectively), and inferior (I1 and I3, respectively) to the foveal center (as indicated in Fig. 1). The mean Sf-CVI was calculated as the average value of horizontal and vertical CVIs, including Sf1-CVI(r = 1 mm) and Sf3-CVI(r = 3 mm).

### Statistical analyses

CVI was compared across different age groups (i.e., 5–9 years, 10–13 years, and 14–18 years), axial lengths (i.e., 21.00–25.00 mm and 25.01–29.00 mm), and myopia groups (i.e., non-myopia(≥ − 0.25D), low myopia(− 0.25D ~ -3.0D), moderate myopia (3.0D ~ -6.0D) and high myopia (≤ − 6.0D)). All data were analyzed to determine the relationship between these factors and CVI. Statistical analyses were conducted using IBM SPSS Statistics version 21.0 (IBM Corp., Armonk, NY, USA). Normality data distribution was tested with the Kolmogorov-Smirnov test. Normally distributed data were expressed as means±standard deviations (SDs). Multiple linear regression was applied to examine the associations among age, AL, spherical equivalent (SE), and CVI of different locations. A value of *p* < 0.05 was considered statistically significant in all analyses.

## Results

### Descriptive statistics analysis results

CVI was measured in 164 of 172 eyes; eight eyes were excluded due to poor image quality. Since 27 younger patients had difficulty cooperating for the GAT examination, we finally obtained reliable GAT-IOP data from 111 eyes of 58 patients. The age of the volunteers ranged from 5 to 18 years with a mean value ± standard deviation (SD) of 10.05 ± 2.65 years. Mean SE was − 1.90 ± 2.48D (ranging from − 10.75D to 6.75D). Mean AL was 24.34 ± 1.29 mm (ranging from 21.50 mm to 27.94 mm). Mean GAT-IOP was 16.99 ± 3.37 mmHg (Table 1). The mean CT in the central foveal was 269.87 ± 63.32 μm (ranging from 93.00 μm to 443.00 μm). The mean Sf-CVI in *r* = 1 mm region to the central foveal was 67.65 ± 2.42% (ranging from 61.49 to 77.27%). The mean Sf-CVI in *r* = 3 mm region to the central foveal was 67.68 ± 1.98% (ranging from 62.63 to 74.13%) (Table 2).

**Table 1:** Demographics, clinical and choroidal characteristics of study subjects (*n* = 164)

Variables	Mean ± SD	Number (%)
Age(years)	10.05 ± 2.65	85
5–9	7.88 ± 1.17	42 (49.41%)
10–13	11.49 ± 1.01	35 (41.18%)
14–18	15.25 ± 1.39	8 (9.41%)
Gender		85
Male	–	45(52.94%)
Female	–	40(47.06%)
AL (mm)	24.34 ± 1.29	164
≤ 25	23.70 ± 0.75	119(72.56%)
>25	26.11 ± 0.66	45(27.43%)
SE (diopter)	−1.90 ± 2.48	164
≥ −0.25	0.84 ± 1.67	33(20.12%)
-0.25 ~ −3.0	−1.49 ± 0.69	94(57.32%)
-3.0 ~ −6.0	−4.02 ± 0.72	23(14.02%)
≤ −6.0	−7.66 ± 1.32	14(8.54%)
GAT-IOP (mmHg)	16.99 ± 3.37	111 (67.68%)

Data presented are means ± standard deviations (SD), except for gender, which is number (%)

*AL* Axial length, *SE* Spherical equivalent, *SFCT* subfoveal choroidal thickness, *GAT-IOP* Goldmann applanation tonometry-intraocular pressure

**Table 2:** Choroidal characteristics of study subjects (*n* = 164)

Variables	Mean ± SD	Range
SFCT (μm)	269.87 ± 63.32	93.00 to 443.00
*r* = 1 mm		
Horizontal CVI (%)	67.99 ± 2.69	61.49 to 78.72
Vertical CVI (%)	67.36 ± 2.65	60.40 to 75.83
Mean CVI (%)	67.65 ± 2.42	61.49 to 77.27
Temporal (%)	67.92 ± 3.00	58.93 to 77.83
Nasal (%)	68.05 ± 3.34	60.84 to 79.60
Superior (%)	67.45 ± 2.90	61.35 to 75.70
Inferior (%)	67.27 ± 3.00	57.84 to 78.46
*r* = 3 mm		
Horizontal CVI (%)	67.97 ± 2.40	59.98 to 75.47
Vertical CVI (%)	67.44 ± 2.08	60.65 to 73.05
Mean CVI (%)	67.68 ± 1.98	62.63 to 74.13
Temporal (%)	67.32 ± 5.74	58.51 to 75.82
Nasal (%)	68.22 ± 2.95	61.30 to 76.09
Superior (%)	67.47 ± 2.35	61.22 to 73.38
Inferior (%)	67.42 ± 2.31	60.08 to 73.06

*SFCT* sub-foveal choroidal thickness, *CVI* choroidal vascularity index

### Choroidal remodeling distribution pattern

The mean CVI of the four regions above was compared in the different myopia groups, respectively. In high myopia group, the measurements of CVI can be ordered as N-CVI (70.17, *r* = 1; 69.54, *r* = 3), T-CVI (70.09, *r* = 1; 69.08, *r* = 3), I-CVI (68.75, *r* = 1; 68.62, *r* = 3) and S-CVI (67.95, *r* = 1; 68.12, *r* = 3). In general, regardless of *r* = 1 mm or *r* = 3 mm, the distribution pattern of CVI was similar. Remarkably, irrespective of the group, the CVI of the horizontal meridian was consistently greater than that of the vertical meridian, even though the difference was not significant (*p*>0.05). In all groups, the mean N-CVI was always the greatest except the low myopia group in which T-CVI had the greatest value. However, One-way *ANOVA* analysis showed no significant differences in CVI of four regions in either myopia group (*p*>0.05).

### CVI in the horizontal meridian underwent the largest change as myopia worsened

**Fig. 2:**
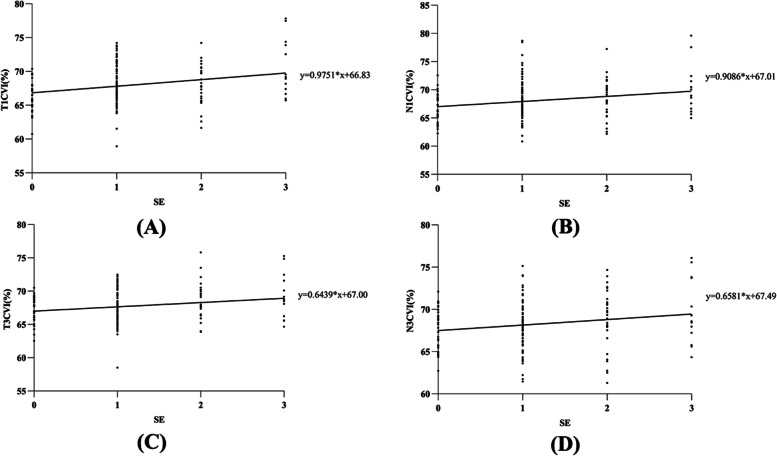
Graph showing the relationship between CVI and degree of myopia. **A** T1- CVI was positively correlated with the degree of myopia (y = 0.9751*X + 66.83, *p* < 0.05, *r*^2^ = 0.089). **B** N1- CVI was positively correlated with the degree of myopia (y = 0.9086*X + 67.01, *p* < 0.05, *r*^2^ = 0.045). **C** T3- CVI was positively correlated with degree of myopia (y = 0.6439*X + 67.00, *P* < 0.05, r2 = 0.045). **D** N3- CVI was positively correlated with degree of myopia (y = 0.6581*X + 67.01, *P* < 0.05, r2 = 0.028)

**Fig. 3:**
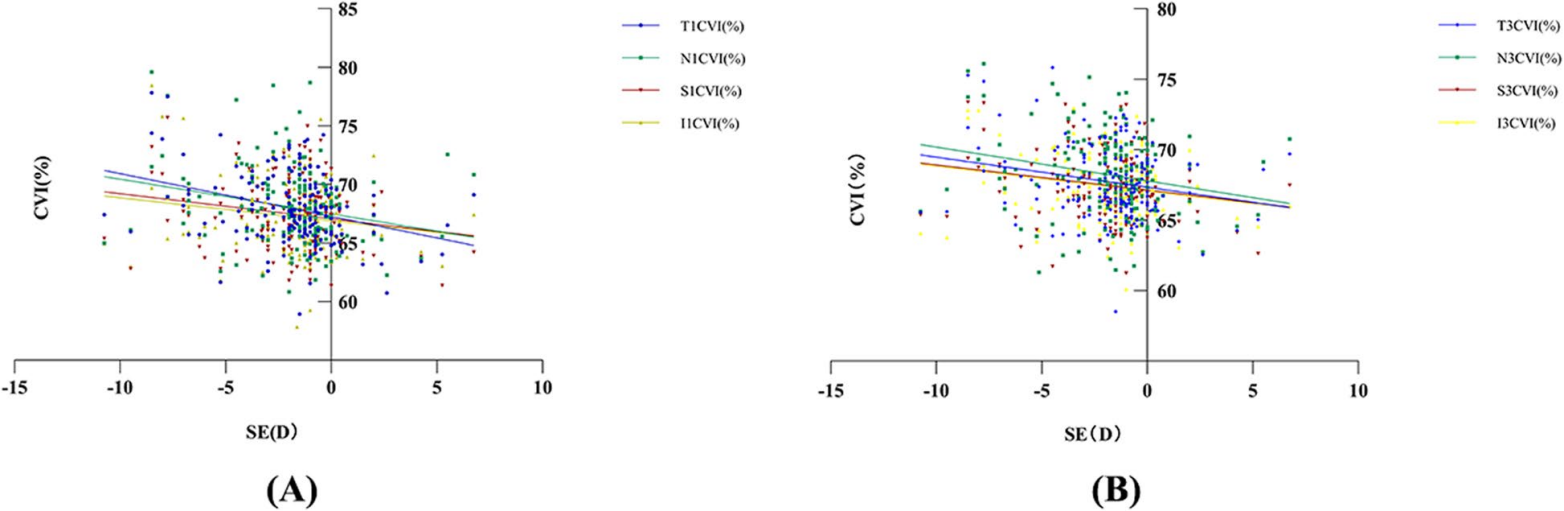
Graph showing the relationship between choroidal vascularity index (CVI) and spherical equivalent (SE). All data points were used in simple linear regressions analysis. Different colored lines represent different regions’ CVI. **A** reveals a negative linear relation between SE and CVI (*r* = 1 mm). **B** shows a negative linear association between SE and CVI (*r* = 3 mm)

Temporal and nasal CVI within the *r* = 1 mm and *r* = 3 mm subfoveal range were positively associated with degree of myopia in young patients (Table 3, Fig. 2). Multiple linear regression results revealed significant correlations between SE and T1-CVI (*p* < 0.05, *r*^2^ = 0.082, β = 0.194), N1-CVI (*p* < 0.05, *r*^2^ = 0.039, β = 0.212) (Table 4). Simple linear regression results revealed that mean Sf1-CVI (*p* < 0.05, *r*^2^ = 0.08) and Sf3-CVI (*p* < 0.05, *r*^2^ = 0.07) were negatively correlated with SE; T1-CVI (*p* < 0.05, *r*^2^ = 0.09) and T3-CVI (*p* < 0.05, *r*^2^ = 0.05) were negatively correlated with SE; N1-CVI (*p* < 0.05, *r*^2^ = 0.05) and N3-CVI (*p* < 0.05, *r*^2^ = 0.04) were negatively correlated with SE (Fig. 3). Notably, there was no significant association between GAT-IOP and CVIs of different regions using a simple linear regression analysis (*p* > 0.05). When we analyzed the relationship between CVI and CT, we found no clear relationship between SFCT and Mean CVI (*r* = 1 mm or *r* = 3 mm) using linear regression (*p* > 0.05).

**Table 3:** Results of simple linear regression analyses between different SE groups and CVI

CVI	Beta	*p*-value	*R*-square
T1CVI N1CVI S1CVI	0.270 0.228 0.132	0.000 0.003 0.093	0.073 0.052 0.017
I1CVI	0.130	0.097	0.017
T3CVI N3CVI S3CVI	0.213 0.184 0.145	0.005 0.016 0.063	0.045 0.028 0.021
I3CVI	0.150	0.055	0.023

SE is treated as ordinal categorical variable. The ordinal categorical values are as follows:≥ − 0.25D = 0;-0.25D ~ -3.0D = 1;-3.0D ~ -6.0D = 2; ≤ − 6.0D = 3

**Table 4:** Multiple linear regression analyses for age, AL and SE as correlates of CVI (*r* = 1 mm)

CVI	Variables	Beta	*p*-value	Adjusted *R*-square
T1CVI	Age	0.013	0.878	
	AL	0.118	0.234	0.082
	SE	0.194	0.044	
N1CVI	Age	−0.084	0.308	
	AL	0.059	0.557	0.039
	SE	0.212	0.030	
S1CVI	Age	−0.048	0.571	
	AL	0.013	0.900	0.001
	SE	0.351	0.181	
I1CVI	Age	−0.107	0.204	
	AL	0.111	0.286	0.011
	SE	0.085	0.401	

All the covariates presented are treated as ordinal categorical variables. The ordinal categorical values are as follows: (1) Age:5-9 yrs. = 0;10-13 yrs. = 1;14-18 yrs. = 2; (2)AL:≤25 mm = 0;> 25 mm = 1; (3) ≥ −0.25D = 0; − 0.25D ~ -3.0D = 1;-3.0D ~ -6.0D = 2; ≤ − 6.0D = 3

### CVI was a more stable measure index than CT

For the CVI of different regions, all the coefficients of variation were less than 5%. In contrast, the coefficient of variation of CT was greater than 20%, which was more than four times higher compared to the same index of CVI. With adjusted SE and AL, there was no significant difference in mean Sf-CVI across age groups (*p* > 0.05). There was, however, a significant difference in choroidal thickness (CT) across different age groups (*p* < 0.05).

## Discussion

Our study explored the relationship between the distribution pattern of choroidal remodeling and the degree of myopia in young patients (aged 5–18 years). Previous research has reported that the remodeling of the choroid is the key factor of pathological changes in high myopia. CT has been wildly used as a vital predictor of choroidal remodeling in highly myopic eyes. This study found CVI to be a more robust measure index than CT. Mean Sf-CVI was not affected by age. There was, however, a significant difference in CT across different age groups.

There is a lot of researches focus on the distribution pattern of CT in highly myopic eyes. As in adults, there is a significant negative correlation between CT and axial growth rate in children [19, 20]. Myopia in children is mainly due to the axial elongation of the eye. In a few cases, it can also be related to the change in corneal refractive power. Children with slow eye growth have significantly thicker choroids, while children with faster eye growth (i.e., those who are developing myopia or whose myopia is developing rapidly) do not show such growth over time, and in many cases, the choroid is thinner [4]. However, some studies have also mentioned that children’s myopia drift is independently related to the growth of the ophthalmic axis and the thinning of the choroid, and the increase of the ophthalmic axis has nothing to do with the decrease of choroid thickness. The thinning of the choroid is more than the secondary stretching effect of eye elongation [21]. In a healthy eye, the choroid is the thickest in the fovea. While in highly myopic eyes, the choroidal thickness of the temporal region may exceed that of the fovea, and the high occurrence of posterior sclera staphyloma in high myopia may be the main reason for choroid morphology. However, study [22] also found that the temporal choroid may have a greater thickness than the fovea choroid even in eyes without posterior sclera staphyloma, suggesting that relative temporal choroid thickening may involve changes in peripheral blood vessels around the optic papilla and a disproportionate temporal shift in the choroid/sclera ratio towards retinal displacement during myopia progression. In both healthy and highly myopic eyes, the lateral choroid of the nose was the thinnest in most studies [23–25]. In addition, Lee et al. [24], after using OCT to measure and evaluate the variation trend of choroid thickness, proposed the temporal choroid thickness/fovea choroid thickness (CTT/CTF) ratio index. It was found that the CTT/CTF ratio index was positively correlated with AL (*p* = 0.031) and the width of peripapillary atrophy (PPA) of the optic disc atrophy arc (*p* = 0.003) but negatively correlated with SE (*p* = 0.012).

CVI is a promising new parameter to assess the remodeling of choroid vessels and retinal blood supply in the macular area. In a study with a sample of 345 healthy eyes, a higher CVI is found to be significantly correlated with thicker SFCT. Unlike SFCT, CVI is not affected by most physiological variables, including AL, IOP, age, gender, blood pressure, and body mass index [16]. Another recent study also reported no significant correlation of CVI with age is found in healthy eyes [26]. Our present findings were broadly consistent with these studies. We did not detect any significant relationship between the CVI and a multitude of physiological factors (i.e., age, AL, and IOP). Moreover, the current study confirmed the finding from a previous study [16] that the coefficient of variation of SFCT was much higher in comparison with CVI. All studies verify the fact that CVI is seen to be robust to other physiological parameters variation. Nevertheless, our study suggested no intrinsic connection between SFCT and CVI, which differed from the study by Agrawal et al. [16]. Notably, few prior studies have explored the relationship between CVI and refractive error, while this study detected a significant positive association between the temporal and nasal CVIs and the degree of myopia in young patients. As proposed by Nickla et al., the non-vascular smooth muscle cells mediate the choroidal thinning by contraction [24]. In that case, the dilatation of vessel lumen occurs, resulting in the enlargement of the choroidal luminal area in the OCT image, which means a higher CVI. We hypothesized that with the progression of myopia, the temporal and nasal choroid responded first. The compensatory vasodilation of choroidal vessels of these two regions, which was contributed by the non-vascular smooth muscle, improved blood supply temporarily and caused a higher value of CVI. While beyond a certain limit, the vessels would not dilate anymore, and the reduction of blood flow may occur, followed by more severe consequences.

It has been proposed that the early-onset high myopia (7 to 11 years old) may be attributed to hereditary, with a higher risk of diffuse chorioretinal atrophy or more severe retinal lesions, while acquired high myopia (usually after 11 years old) is often influenced by the environment [27]. Pärssinen et al. [28] conducted a 22-year study by following children participants into their adulthood and found that the most important predictors of high myopia were younger age at baseline and faster myopia development during the first follow-up. High myopia carries a greater risk of ocular diseases such as cataracts, glaucoma, and retinal detachment [29], among which myopic macular degeneration (MMD) is one of the most frequent causes of vision loss or irreversible blindness in developed countries, especially in East Asia with higher myopia rates [30, 31]. Wong et al. [30] found that the degree of choroidal thinning was closely related to the severity of MMD, while scleral thickness was weakly related to MMD; this information suggests that choroidal thinning leads to a reduction in choroid perfusion and choroidal ischemia, with subsequent upregulation of angiogenic factors is important pathogenesis of MMD [32]. Patients with lacquer cracks are likely to be at higher risk of visual impairment and the development of myopic choroidal neovascularization. Some studies believe that choroidal thickness can be used as a biological indicator to predict the occurrence and severity of lacquer cracks in highly myopic eyes [33]. Additionally, myopic choroidal neovascularization has also been confirmed to be closely related to choroidal thinning [34, 35]. CVI is expected to be used together with CT as a biomarker of choroidal blood flow remodeling in the future.

It is worthwhile to mention that there are some limitations in the present study. Firstly, our study was a cross-sectional design, and the sample size was relatively small. Secondly, several younger participants were unable to cooperate with the GAT examination, leading to the loss of IOP data. However, the remaining sample size was sufficient according to previous recommendations on required sample sizes when conducting regression analyses [36]. Thus, we believe the impact of missing data on the results to have been limited. Thirdly, the calculation of CVI is affected by the OCT image quality and manual measurement. If the line of choroidal-scleral interface cannot be determined, the results of CVI would be unreliable. In this work, we excluded the OCT images with unacceptable quality and adopted multiple measurements to ensure the interpretability of results. Our future work will explore automated algorithm to provide more comprehensive information on the choroidal remodeling.

## Conclusion

Temporal and nasal CVIs within the *r* = 1 mm and *r* = 3 mm subfoveal range were positively associated with the degree of myopia in young patients. CVI is the horizontal meridian that underwent the most significant change as myopia worsened. The CVI value may be used as a parameter in evaluating the choroidal vascular status and a superior candidate biomarker for myopic progression. Future research, including studies focusing on choroid changes, is necessary to elucidate the underlying pathophysiology and pathology of myopia.

## Data Availability

The datasets used and analyzed during the current study are available from the corresponding author on reasonable request.

## Abbreviations

D: Diopter
SD-OCT: Spectral-domain optical coherence tomography
EDI: Enhanced depth imaging
CT: Choroidal thickness
CVI: Choroidal vascularity index
N: Nasal
T: Temporal
S: Superior
I: Inferior
AL: Axial length
SE: Spherical equivalent
IOP: intraocular pressure
GAT: Goldmann applanation tonometry
RPE: Retinal pigmented epithelium
NIH: National Institutes of Health
Sf: Subfoveal
SD: Standard deviation
CTT: Temporal choroid thickness
CTF: Fovea choroid thickness
PPA: Peripapillary atrophy
MMD: Myopic macular degeneration
